# Exploring the Biochemical Profile of *Beta vulgaris* L.: A Comparative Study of Beetroots and Swiss Chard

**DOI:** 10.3390/plants14040591

**Published:** 2025-02-14

**Authors:** Daiana Almeida, Spyridon A. Petropoulos, Tayse F. F. da Silveira, Tânia C. S. P. Pires, Isabel C. F. R. Ferreira, Ângela Fernandes, Lillian Barros

**Affiliations:** 1 Centro de Investigação de Montanha, LA SusTEC, Instituto Politécnico de Bragança, Campus de Santa Apolónia, 5300-253 Bragança, Portugal; daiana@ipb.pt (D.A.); tayse.silveira@ipb.pt (T.F.F.d.S.); tania.pires@ipb.pt (T.C.S.P.P.); iferreira@ipb.pt (I.C.F.R.F.); lillian@ipb.pt (L.B.); 2 Department of Agriculture Crop Production and Rural Environment, University of Thessaly, Fytokou Street, 38446 Volos, Greece

**Keywords:** chemical composition, bioactive properties, betalains, phenolic composition, antimicrobial properties, antioxidant activity

## Abstract

In this study, leaves and roots from three beetroot cultivars (cv. Albina Vereduna (white roots), cv. Burpee’s Golden (golden roots), and cv. Pablo F1 (red roots)), as well as Swiss chard leaves (also known as “rhubarb chard”, or *Beta vulgaris* subsp. *cicla* var. *flavescens*) were evaluated in terms of their chemical profile and bioactive properties. Roots were characterized by high carbohydrate content, which also contributed to greater energy values. In contrast, fibers were the predominant macronutrient in leaves, followed by carbohydrates. In both leaves and roots, the most abundant organic acids were quinic and oxalic, while the major free sugar was sucrose. The profile of fatty acid varied between the studied plant parts, with saturated fatty acids prevailing in root samples, while leaves exhibited higher levels of polyunsaturated fatty acids. Regarding phenolic composition, a total of 19 compounds were tentatively identified in leaves (including derivatives of vitexin, isorhamnetin, quercetin, and ferulic, sinapic, and *p*-coumaric acids), while the roots exhibited a less diverse composition, with a total of eight compounds identified (e.g., derivatives of ferulic, sinapic, *p*-coumaric and caffeic acids). A total of eight betalains were also identified, out of which seven were classified as betacyanins and one as betaxanthin. The leaves of Swiss chard presented compounds from both classes, while the roots and leaves of cv. Pablo F1 were characterized only by the presence of betacyanins, and those of cv. Burpee’s Golden only by betaxanthin. All samples exhibited relevant activity against *Y. enterocolitica*, *L. monocytogenes*, and *S. aureus*, although leaf samples demonstrated better antioxidant capacity. In conclusion, beetroot leaves outperformed their corresponding roots in terms of chemical composition, antioxidant, and antimicrobial activity, suggesting their high potential as nutrient-rich and functional ingredients in a diverse and well-balanced diet.

## 1. Introduction

*Beta vulgaris* L. is a biennial herbaceous plant, taxonomically classified in the Amaranthaceae family, according to the Angiosperm Phylogeny Group (APG) system, or to the Chenopodiaceae family, according to the Cronquist system, and in the Caryophyllales order [1]. Divided into three subspecies, the species is composed of two groups depending on the cultivation conditions, e.g., cultivated varieties and wild varieties [2].

The first group of cultivated species includes the main representatives of the species classified in five groups, e.g., table beet (Conditiva group), Swiss chard (Flavescens group), chard or spinach beet (Cicla group), sugar beet (Altissima group) and fodder beet (Crassa group) from which the fleshy edible roots and the leaves are used for culinary purposes, as well as in the industrial sector or as animal feed [3,4]. The second group is composed of the wild maritime beets, represented solely by sea beets (*Beta vulgaris* L. subsp. *maritima*), which are the ancestral form of all the cultivated varieties [4,5].

*Beta vulgaris* L. is a very diverse and versatile species, which probably originated between northern Africa and southern Europe and then spread along the coasts of the Mediterranean and Atlantic seas [6]. Since then, beets and their relatives have been cultivated worldwide for food (human and animal) and medicinal purposes. Ancient records describe recipes from Greek, Roman, and Egyptian civilizations using beetroot (root and leaves) as an ingredient in the preparation of culinary dishes and medicines [7].

As an integral part of culinary traditions in the Mediterranean basin, beetroot nowadays finds its place in a multitude of dishes and preparations, with its roots being consumed raw, roasted, boiled or pickled and used in the preparation of juices, beverages, salads, breads and soups, while its leaves are increasingly used as microgreens or baby leaves for salads [8]. Swiss chard, meanwhile, is highly appreciated for its vigorous leaves and stems [7,9]. Moreover, recent studies suggest the incorporation of beetroot extracts in functional food products due to their bioactive potential [10,11,12].

However, beyond their culinary appeal, beetroot and chard have been gaining attention for being rich and nutritious sources of proteins; dietary fibers; vitamins, such as tocopherols (vitamin E); carotenoids; inorganic nitrates (NO_3_); and essential minerals such as potassium, sodium, phosphorous, calcium, magnesium, copper, iron, zinc, and manganese [6]. Moreover, the remarkable presence of biologically active phytochemicals has attracted the attention of researchers who investigate the promising biological properties related to these compounds and their application in the food and pharmaceutical industries [13]. Namely, the presence of phenolic compounds has been reported, including phenolic acids (e.g., gallic, syringic, and caffeic acids); important flavonoid derivatives (e.g., apigenins); polyunsaturated fatty acids (e.g., α-linolenic and linoleic acids) [5]; and oligosaccharides with prebiotic potential [14]. Moreover, important heterocyclic nitrogen-based and water-soluble pigments such as betacyanins (red–violet color) and betaxanthins (yellow–orange color), part of the betalains family, have been recorded in a limited number of species in the Caryophyllales order and have been suggested as excellent antioxidant and anti-inflammatory agents, among other classes of health-promoting bioactive compounds [2,15,16,17] or as natural colorants with several applications in the food industry [13,18,19]. Recent studies have also revealed compelling evidence that the consumption of beetroots and leaves of Swiss chard are associated with the health promotion of consumers, due to their invaluable biological properties, including antioxidant, anti-hypertensive, and anti-carcinogenic activities, as well as notable hypoglycemic, lipid metabolism regulation hepatoprotective and hypotensive potentials [20,21,22,23,24].

The multifaceted nature of *B. vulgaris* L. makes it a species of major significance, with great interest in its nutritional and bioactive potential. Considering the increasing demand of consumers for functional and health promoting food products, the present study evaluated the nutritional value and chemical composition of beetroot and Swiss chard varieties, aiming to highlight their potential beneficial role in human diet, as well as to capture the variation among the edible parts of different *Beta* cultivars in terms of the tested parameters. Furthermore, the biological activities (antioxidant and antimicrobial) of hydroethanolic extracts of the samples were also assessed.

## 2. Materials and Methods

### 2.1. Samples

The experiment took place at the experimental farm of the university of Thessaly, in Velestino, Greece during the spring–summer period of 2021. Seeds of three beetroot cultivars (e.g., *Beta vulgaris* cv. Albina vereduna, *Beta vulgaris* cv. Burpee’s golden and *Beta vulgaris* cv. Pablo F1), and one Swiss chard variety (*Beta vulgaris* subsp. *cicla* var. *flavescens*) were sown directly in 6 L pots that contained peat and perlite (1:1; *v*/*v*) at the beginning of March 2021. Pots were cultivated outdoors and put on a plastic mulch to prevent weed growth. After seedling emergence, plants were thinned to one plant per pot, while 20 pots were used for each variety (80 pots in total). Plants were fertigated three times throughout the growing period with a nutrient solution that contained 200 mg of N-P-K and trace elements, using 300 mL of nutrient solution each time. Irrigation was applied with a sprinkler irrigation system at regular intervals, depending on the environmental conditions. Harvest took place in the middle of June (14 weeks after sowing) by pulling the plants from the growing substrate for all the tested cultivars. After harvest, plants were rinsed with tap water to remove the remaining substrate from roots, and then leaves were separated from roots. Fresh samples of leaves and roots were put in air-sealed bags and kept under deep-freezing conditions (−80 °C) until lyophilization. The lyophilized samples were ground to a fine powder (approximately 20 mesh) and then put in plastic bags under vacuum and stored under deep-freezing conditions until further analysis.

### 2.2. Extract Preparation

Maceration extracts were made from the lyophilized samples by adding 90 mL of an 80% ethanolic solution to 3 g of each sample. After 1 h of stirring at room temperature, the mixtures were filtered via paper filters. The residues were extracted once more in identical circumstances. After combining the hydroethanolic extracts from the two extractions, the ethanol was evaporated at 40 °C under reduced pressure. The resulting aqueous phases were frozen and freeze-dried to produce dry extracts, which were then utilized to analyze the samples’ biological characteristics and the composition in phenolic compounds.

### 2.3. Proximal Composition

The Official Methods of Analysis of the Association of Official Analytical Chemists (AOAC) [25] were implemented for the determination of proximal composition (proteins, lipids, ash, dietary fibers, carbohydrates, and total energy) of the samples. Protein content was assessed by the macro-Kjeldahl method (conversion factor = 6.25), via acid digestion, distillation, and titration [26]. Crude fat content was estimated after extraction for 7 h with a Soxhlet apparatus using petroleum ether as a solvent. The ash content was determined through incineration at 550 ± 10 °C for a period of 5 h in a muffle furnace. Total dietary fiber (TDF) was assessed with an enzymatic–gravimetric method (TDF-100A Kit; Sigma Aldrich, St. Louis, MO, USA). Total carbohydrates were calculated by their difference, and energy according to the following equation:

Energy (kcal) = 4 × (g_protein_ + g_carbohydrate_) + 9 × (g_fat_).

### 2.4. Chemical Profile of Hydrophilic Compounds

#### 2.4.1. Free Sugars

To determine the free sugar content, 40 mL of ethanol solution (80%) and 1 mL of internal standard (melezitose, 25 mg/mL) were added to 1 g of dry and powdered samples. The extraction was conducted for 1 h and 30 min at 80 °C in a water bath. After the extraction, the mixtures were filtered through Whatman N° 4 paper filters (Sigma Aldrich, St. Louis, MO, USA), and the ethanol was evaporated at 40 °C. After concentration, the aqueous portions obtained were dissolved in distilled water up to a final volume of 5 mL, then filtered with 0.2 µm nylon filters into vials, and finally were analyzed by high performance liquid chromatography (HPLC) at 35 °C using an HPLC system (Knauer, Smartline system, Berlin, Germany) equipped with a refraction index (RI) detector (Knauer Smartline 2300, Berlin, Germany) and a 100-5 NH2 Eurospher column (4.6 × 250 mm, 5 mm, Knauer; Berlin, Germany). The extraction method, mobile phase and the identification procedure used has been described by Spréa et al. [27].

#### 2.4.2. Organic Acids

Following the methods detailed by Barros et al. [28], the organic acids were extracted by stirring 1 g of each sample with 25 mL of metaphosphoric acid at room temperature for 25 min. The extracts were then filtered with paper filter, and subsequently through 0.2 μm nylon filters into vials. The assessment of organic acids composition was performed by ultra-fast liquid chromatography equipped with a reverse phase column C18 SphereClone (Phenomenex, 5 μm, 250 × 4.6 mm; Phenomenex, Alcobendas, Spain), thermostated at 35 °C and coupled to a diode array detector (UFLC-DAD; Shimadzu Cooperation, Kyoto, Japan). The samples were eluted with 3.6 mM sulfuric acid solution, using a flow rate of 0.8 mL/min, and a wavelength of 215 nm to detect the organic acids. The identified organic acids were further quantified based on the comparison of chromatograms with commercial standards.

### 2.5. Chemical Profile of Lipophilic Compounds

#### 2.5.1. Fatty Acids

The profiles of fatty acid methyl esters (FAMEs) were determined from the lipid fraction previously obtained in the fat determination, which was subjected to a derivatization by transesterification reaction, according to the methodology previously described by Obodai et al. [29]. The fatty acid profile was determined by gas chromatography (GC) with flame ionization detection (FID), using a YOUNG IN Chromass 6500 GC System instrument (YOUNG IN; Anyang-si, Republic of Korea) equipped with a split/splitless injector. Fatty acid identification and quantification were made by comparing the relative retention times of FAME peaks from samples with standards (standard mixture 47885-U, Sigma, St. Louis, MO, USA). The results were recorded and processed using Clarity 4.0.1.7 software (DataApex, Prague, Czech Republic), and expressed in the relative percentage (%) of each fatty acid.

#### 2.5.2. Tocopherols

Tocopherols were extracted following the procedure previously described by Spréa et al. [27]. The extract obtained was dried using a stream of nitrogen, and it was subsequently redissolved in 2 mL of hexane, filtered through 0.2 μm nylon filters into amber vials, and analyzed by HPLC. For chromatographic analysis, an integrated quaternary pump system (Knauer, Smartline 1000 system; Knauer GmbH, Berlin, Germany), a degasser (Smartline 5000; Knauer GmbH, Berlin, Germany), an autosampler (AS-2057 2500; Jasco International, Tokyo, Japan), and a fluorescence detector (FL; Jasco; Jasco International, Tokyo, Japan) programmed for excitation at 290 nm and emission at 330 nm were used. Separation of compounds was performed using a polyamide II normal phase column (5 μm, 250 × 4.6 mm, WMC Waters, Japan) operating at 30 °C (Jones 7971 R Grace oven; Berlin, Germany). The mobile phase used was a hexane/ethyl acetate mixture (70:30, *v*/*v*) with a flow rate of 1 mL/min. Data were analyzed using Clarity 2.4 software (DataApex, Prague, Czech Republic). Quantification was based on the fluorescence signal response using the PI method and by chromatographic comparison with standards. Results are expressed as mg per 100 g dw.

### 2.6. Betalains

The extraction of samples (500 mg) was performed with 5 mL of ethanol:water solution (50:50, *v*/*v*) with 1.5% of citric acid, under constant magnetic agitation at 50 °C for 40 min. The mixtures were then filtered through paper filters and the residues were extracted once more, at similar conditions, following the protocol of Lazăr et al. [30], after slight changes. Subsequently, the ethanol was evaporated at 40 °C, and the aqueous phases obtained were made up to a final volume of 5 mL, filtered through 0.2 µm nylon filters for subsequent chromatographic analysis using a Dionex Ultimate 3000 UPLC (Thermo Fisher Scientific, San Jose, CA, USA) coupled to a DAD.

The chromatographic separation was performed using a Waters Spherisorb S3 ODS-2 C18 column (3 µm, 4.6 mm 150 mm; Waters, Milford, MA, USA), thermostated at 35 °C. The solvents used were (A) 0.1% trifluoroacetic acid (TFA) in water and (B) acetonitrile and the sample injection volume was 50 μL. The elution gradient applied was 100% A for 15 min, 100 to 90% B for 5 min, 85% B for 5 min, 85 to 82% B for 5 min, 50% B for 7 min, and 50 to 100% B for 13 min, using a flow rate of 0.5 mL/min. MS detection was performed using an Ion Trap Linear LTQ XL mass spectrometer (Thermo Fisher Scientific, San Jose, CA, USA), with an electrospray ionization (ESI) source. The system operated with a spray voltage of 4.8 V at 325 °C and a capillary voltage of 39 V. The collision energy used was 35 (arbitrary units), and the results obtained were analyzed using the Xcalibur^®^ program (Thermo Fisher Scientific, San Jose, CA, USA). For identification, the betalain extracts of each sample were measured in the spectral range of maximum absorbances of betaxanthins and betacyanins at λ = 480 nm and λ = 530 nm, respectively, and characterized according to the UV and mass spectra and retention times. For the quantitative result, a calibration curve was obtained after reading the absorbance of known concentrations of compounds, and the compounds quantified using the UV–vis signal and maximum absorbance of commercial standards and, when not available, using similar compounds. The results are expressed in mg per g of dw.

### 2.7. Phenolic Compounds

The phenolic composition of the maceration extracts was determined via high performance liquid chromatography (HPLC), performed on a Dionex Ultimate 3000 UPLC (Thermo Fisher Scientific, San Jose, CA, USA) equipped with a diode array detector (DAD), following a previously published procedure [31]. A Waters Spherisorb S3 ODS-2 C18 column (3 μm, 150 × 4.6 mm, Waters, Milford, MA, USA) was used for the separation, operating at 35 °C. The mobile phase was 0.1% formic acid (A) and acetonitrile (B). The elution gradient was 15% B (5 min), 15 to 20% B (5 min), 20 to 25% B (10 min), 25 to 35% B (10 min), and 35 to 50% B (10 min). Mass detection was carried out using an LTQ Orbitrap XL mass spectrometer (Thermo Scientific, San Jose, CA, USA), with an ESI electrospray ionization source. The system operated at a spray voltage of 5 kV at 325 °C with a capillary voltage of −20 V. The spectra were recorded in negative ion mode between 100 and 1500 *m*/*z*. The results were collected and analyzed using the Xcalibur^®^ program (version 2.2.0.48; Thermo Fisher Scientific, San Jose, CA, USA). The phenolic compounds present in the samples were characterized by comparison between their UV-vis spectra, mass spectra and retention times with standards and literature, when available. For the quantitative analysis, a calibration curve was obtained by injecting known concentrations of different standards, and the compounds quantified using the UV–vis signal of the commercial standards at their maximum absorbance as a basis and, when not available, from other compounds with the same phenolic group. The results are expressed in mg per g of extract.

### 2.8. Bioactive Properties

#### 2.8.1. Antioxidant Activity

The evaluation of the antioxidant potential was performed in vitro on the previously prepared hydroalcoholic extracts using the thiobarbituric acid reactive substances (TBARS) lipid peroxidation inhibition assay, based on the method described by Sarmento et al. [32]. Briefly, porcine (*Sus scrofa*) brains were obtained from official slaughtering animals, dissected and homogenized with a polytron in ice-cold Tris–HCl buffer (20 mmol/L, pH 7.4) to produce a 1:2 (*w*/*v*) brain tissue homogenate, which was centrifuged at 3000× *g* for 10 min. An aliquot (0.1 mL) of the supernatant was incubated with the different solution concentrations (0.2 mL) in the presence of FeSO_4_ (10 μmol/L; 0.1 mL) and ascorbic acid (0.1 mmol/L; 0.1 mL) at 37 °C for 1 h. The reaction was stopped by the addition of trichloroacetic acid (28% *w*/*v*, 0.5 mL), followed by thiobarbituric acid (TBA, 2%, *w*/*v*, 0.38 mL), and the mixture was then heated at 80 °C for 20 min. After centrifugation at 3000× *g* for 10 min to remove the precipitated protein, the color intensity of the malondialdehyde (MDA)–TBA complex in the supernatant was measured by its absorbance at 532 nm. The inhibition ratio (%) was calculated using the following formula: inhibition ratio (%) = [(A − B)/A] × 100%, where A and B are the absorbance of the control and the compound solution, respectively. The extract concentration, providing 50% of antioxidant activity (EC_50_), was calculated from the graph of TBARS formation inhibition against extract concentrations. Trolox was used as a standard.

#### 2.8.2. Antimicrobial Activity

The antibacterial properties were determined following the rapid colorimetric method by microdilution and using the *p*-iodonitrotetrazolium chloride (INT) dye, as provided by Pires et al. [33]. Three gram-positive bacteria were tested: *Staphylococcus aureus* (ATCC 25923), *Bacillus cereus* (ATCC 11778) and *Listeria monocytogenes* (ATCC 19111), as well as 5 gram-negative bacteria: *Escherichia coli* (ATCC 25922), *Salmonella enterocolitis* (ATCC 13076), *Pseudomonas aeruginosa* (ATCC 9027), *Yersinia enterocolitica* (ATCC 8610) and *Enterobacter cloacae* (ATCC 49741), all of which are food isolates. All of these microorganisms were purchased at Frilabo, Porto, Portugal. The samples were first dissolved in 5% (*v*/*v*) dimethyl sulfoxide (DMSO) and 95% of autoclaved distilled water to obtain a final concentration of 20 mg/mL for the stock solution. An amount of 90 μL of this concentration was added in the first well (96-well microplate) in duplicate with 100 μL of tryptic soy broth (TSB). In the remaining wells 90 μL of TSB medium were added. Then the samples were serially diluted to obtain the concentration ranges (10 to 0.03125 mg/mL). To finish, 10 μL of inoculum (standardized at 1.5 × 10^6^ colony forming units (CFUs)/mL) was added to each of the wells, assuring the presence of 1.5 × 10^5^ CFU. Two negative controls were prepared, one with TSB and another one with the extract. Two positive controls were prepared with TSB and each inoculum and another with medium, antibiotics, and bacteria. Ampicillin and streptomycin were used for all bacteria tested and meticilin was also used for *S. aureus*. The microplates were incubated at 37 °C for 24 h. The minimum inhibitory concentration (MIC) of the samples was detected following the addition (40 μL) of 0.2 mg/mL INT and incubation at 37 °C for 30 min. MIC was defined as the lowest concentration to inhibit the visible bacterial growth, determined by a change in the coloration from yellow to pink if the microorganisms are viable. For the determination of minimum bactericidal concentration (MBC), 10 μL of liquid from each well that showed no change in color was plated on solid medium and blood agar (7% sheep blood) and incubated at 37 °C for 24 h. The lowest concentration to yield no growth determined the MBC, with the MBC defined as the lowest concentration required to kill bacteria.

Antifungal activity was assessed using the method of Heleno et al. [34], and the fungi used were *Aspergillus fumigatus* (ATCC 204305) and *A. brasiliensis* (ATCC 16404). Ketoconazole (Frilabo, Porto, Portugal) was implemented as a positive control. The antifungal activity was performed according to the procedure described by Heleno et al., 2013.

The micromycetes were maintained on malt agar and the cultures stored at 4 °C and were further placed in new medium and incubated at 25 °C for 72 h. In order to investigate the antifungal activity, the fungal spores were washed from the surface of agar plates with sterile 0.85% saline containing 0.1% Tween 80 (*v*/*v*). The spore suspension was adjusted with sterile saline to a concentration of approximately 1.0 × 10^5^ in a final volume of 100 μL per well. The samples were first dissolved in 5% (*v*/*v*) DMSO and 95% autoclaved distilled water to obtain a final concentration of 10 mg/mL for the stock solution. Afterwards, 90 μL of this concentration was added in the first well (96-well microplate) in duplicate with 100 μL of malt extract broth (MEB).

In the remaining wells, 90 μL of medium MEB were placed. Then the samples were serially diluted to obtain the concentration ranges (10 to 0.03125 mg/mL). MIC determinations were performed by a serial dilution technique using 96-well microplate. The lowest concentrations without visible growth (with a binocular microscope) were defined as MICs. The fungicidal concentration (MFC) was determined by serial subcultivation of 2 μL of tested compounds dissolved in medium and inoculated for 72 h, into microplates containing 100 μL of MEB per well and further incubated for 72 h at 26 °C. The lowest concentration with no visible growth was defined as MFC, indicating 99.5% killing of the original inoculum. Commercial fungicide ketoconazole (Frilabo, Porto, Portugal) was used as positive control.

### 2.9. Statistical Analysis

All analyses were conducted in triplicate, and the results are presented as mean ± standard deviation (SD) (except for antimicrobial activity). Statistical tests were performed using SPSS statistics software (IBM SPSS Statistics for Windows, Version 25.0; IBM Corp., Armonk, NY, USA). The results were analyzed with a one-way analysis of variance (ANOVA) followed by a means comparison with Tukey’s honestly significant difference (HSD) test at *p* = 0.05. A Student’s *t*-test was used (*p* = 0.05) when only two samples were assessed.

## 3. Results and Discussion

### 3.1. Proximal Composition

The results presented in Table 1 show the proximal composition of the tested leaf and root samples referring to the content of lipids, proteins, ash, fiber, carbohydrates, and energy value.

In root samples, carbohydrates were the main macronutrients found (68.3 to 70.6 g/100 g dw), followed by dietary fibers (19.5 to 20.9%), proteins (5.46 to 7.32 g/100 g dw), ash (2.35 to 4.0 g/100 g dw) and lipids. On the other hand, leaf samples of beetroots had a high content of carbohydrates and dietary fibers (25.5 to 37.8 g/100 g dw and 33.9 to 37.3 g/100 g dw, respectively), followed by ash (13.46 to 20.99 g/100 g dw), proteins (11.84 to 12.23 g/100 g dw) and lipids (3.28 to 4.01 g/100 g dw). In the case of Swiss chard leaves, carbohydrates were the prevalent macronutrient (42.9 g/100 g dw), followed by carbohydrates (33.1 g/100 g dw), ash (11.91 g/100 g dw), proteins (9.94 g/100 g dw) and lipids (2.18 g/100 g dw). Among all of the samples studied, the roots presented high energy values (347.0 to 354.4 kcal/100 g dw), a finding which is closely related to the high levels of carbohydrates recorded in these plant parts. The leaves of cv. Pablo F1 stood out for their high dietary fiber, ash and lipid contents (37.3 g/100 g dw, 20.99 g/100 g dw and 4.01 g/100 g dw, respectively), while the highest carbohydrate content was recorded in the leaves of cv. Albina Vereduna and cv. Burpee’s Golden (68.3 and 19.9 g/100 g dw, respectively), thus having a lower energy value. Similarly, the roots of cv. Albina Vereduna had the highest energy value.

Lucky et al. [35] evaluated the proximal composition of beetroot powder to be applied in the production of cakes, and the results obtained were similar to those found in the present study regarding the ash content (3.57 g/100 g dw), lipids (1.58 g/100 g dw) and proteins (13.01 g/100 g dw), although that study reported a higher total dietary fiber content (55.18%) than our study. Moreover, Kohajdová et al. [36] analyzed the ash, protein, and total dietary fiber composition of root powder from a red beet variety (*B. vulgaris* cv. Betina) aiming to incorporate it into bakery products. The results obtained demonstrate that the studied beet variety presented protein (10.71 g/100 g dw) and ash (4.12 g/100 g dw) levels similar to those of the varieties evaluated in the present work, whereas the fiber content was higher than our study (65.71%, compared with the 19.9% of our study).

Asadi and Khan [37] evaluated the proximal composition of the powder prepared from dried beet leaves to be incorporated into cookies and found similar results to the present study regarding fiber content (35.98%), carbohydrates (46.43%), lipids (2.23%), ash content (22.29%) and energy value (302.90 kcal/100 g dw), whereas proteins content was higher than our study (24.27 g/100 g dw). In the case of Swiss chard leaves, Abdi et al. [38], who evaluated samples collected from local producers in western Ethiopia, also found similar values for carbohydrates (44.95 g/100 g dw), lipid content (1.75 g/100 g dw) and energy value (316.41 kcal/100 g dw). In contrast, Swiss chard leaves of that study had lower ash (2.66 g/100 g dw) and total dietary fiber content (11.49%), and higher protein content (30.19 g/100 g dw) than the present work. According to the literature, a significant variability was recorded in macronutrients composition in the roots of different red beetroot genotypes, as well as between the different root parts (skin and flesh of the root) [39].

Therefore, it could be suggested that a high variability is present among the beetroot germplasm in terms of proximal composition, while other parameters, such as growing conditions and agronomic practices, should be also considered to justify the existing variability. Moreover, the results of the present work suggest that, although the consumption of beetroot leaves is not as popular as that of Swiss chard, they could be a valued dietary option to consider due to their excellent nutritional properties.

### 3.2. Chemical Composition Regarding Hydrophilic Compounds

#### 3.2.1. Free Sugars

Free sugars were also analyzed, and the results are presented in Table 1. Among all of the samples, the roots stood out for their high sugar content, with only two being identified—sucrose being the prevalent sugar (3.94 to 40.79 g/100 g dw), followed by trehalose (0.38 to 0.70 g/100 g dw). On the other hand, in the case of leaves, sucrose (3.24 to 6.21 g/100 g dw) and glucose (2.24 to 5.65 g dw) were the main detected sugars, followed by fructose (0.45 to 2.8 g/100 g dw) and trehalose (0.41 to 0.82 g/100 g dw). In general, roots had a higher sugar content than leaves, with cv. Pablo F1 roots being the sample with the highest total sugars content (41.5 g/100 g dw) due to the high content of sucrose (40.8 g/100 g dw). On the other hand, the highest glucose and fructose content was recorded for the leaves of cv. Albina Vereduna (5.65 and 2.8 g/100 g dw, respectively), while trehalose content was the highest in the leaves of cv. Burpee’s Golden.

Similar to our study, Wruss et al. [40], who analyzed the biochemical composition of juice prepared from seven red-colored beet varieties grown in Austria (Ägyptische Plattrunde, Bolivar, Forono, Mona Lisa, Moronia, Redval and Robuschka), found that, in all varieties evaluated, sucrose was the most abundant sugar, followed by glucose. However, in that study the authors also identified low amounts of fructose, which is not recorded in the present work. Gruska et al. [41] also determined the carbohydrate content in sugar beet roots immediately after harvest and after 3 months of storage with and without protective covering, suggesting that sucrose was the predominant sugar in all the samples regardless of the treatment. In the same study—with the exception of sucrose fructose—glucose, galactose, raffinose and trehalose were also identified, though in lesser amounts. These results suggest that roots of beets are a rich source of free sugars, with sucrose being the most abundant, while other sugars can be detected in lesser amounts depending on the genotype and the experimental conditions.

Regarding Swiss chard, Mzoughi et al. [42] also identified sucrose as the main sugar (16.8 g/100 g dw) in wild chard leaves harvested in Tunisia, followed by glucose (4.29 g/100 g dw). Fructose (0.69 g/100 g dw), galactose (0.57 g/100 g dw), arabinose (0.045 g/100 g dw), rhamnose (0.27 g/100 g dw) and raffinose (0.92 g/100 g dw) were detected in lower amounts and no trehalose was identified.

#### 3.2.2. Organic Acids

In all of the samples studied, six organic acids were identified and quantified, namely succinic acid, which was the most abundant in all of the cultivars and plant parts, followed by oxalic, quinic, malic, citric acids and, finally, fumaric acid, which was present only in trace amounts (Table 1). In general, the leaves had higher levels of individual organic acids than the roots, except for succinic acid, where the root content was higher than leaves in the cases of cv. Albino Vereduna and Pablo F1 (6.0 and 15.0 g/100 g dw, respectively). Swiss chard had high levels of succinic acid (12.4 g/100 g dw), followed by lower levels of quinic (2.54 g/100 g dw), oxalic (2.37 g/100 g dw), citric (2.2 g/100 g dw) and malic acids (1.4 g/100 g dw), while traces of trehalose were also detected. Regarding the different varieties, cv. Pablo F1 recorded the highest content of individual and total organic acids, either in leaves or in roots.

Bavec et al. [43] evaluated the influence of five different cultivation systems (conventional, integrated, organic, biodynamic and control) on the composition of the beetroot (cv. Rote Kugel). The authors suggest the prevalence of citric acid, followed by shikimic acid, and finally, malic and fumaric acids, which were detected at the lowest levels. Sagardoy et al. [44] determined the influence of treatments with different concentrations of zinc on the profile of organic acids in the leaves and roots of sugar beet (*B. vulgaris* L. cv. Orbis) and they verified the prevalence of oxalic and malic acids, whereas, in contrast to our study, succinic acid was the acid with the lowest content. Although oxalic acid is a component of many foods, due to its anti-nutritive properties it can pose potential health risks. In particular, it can reduce the bioavailability of other nutrients and increase the formation of kidney stones [4]. Therefore, given that beetroot is known for its high levels of oxalates, its moderate consumption is recommended. This is especially so for the leaves which recorded higher amounts of oxalic acid than roots in all of the beetroot samples, as well as the leaves of Swiss chard, where amounts similar to beetroot leaves were detected. According to the literature, the soluble and insoluble oxalate contents in the leaves of beetroot and Swiss chard were considerably higher when compared with the roots [45], a finding which is consistent with the results obtained in the present work.

### 3.3. Chemical Composition of Lipophilic Compounds

#### 3.3.1. Fatty Acids

**Figure 1:**
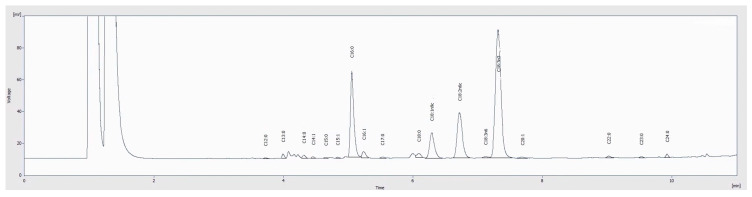
Fatty acid profile of Swiss chard leaves (*B. vulgaris* subsp. *cicla* var. *flavescens*) analyzed with GC-FID.

The evaluation of the fatty acid profile allowed the identification of 21 compounds, as described in Table 2, where the most abundant fatty acids were palmitic acid (C16:0), α-linolenic acid, (C18:3n3), linoleic (C18:2n6c), and oleic (C18:1n9c) acids with amounts that varied among the tested samples. The roots of cv. Burpee’s Golden were the most abundant in palmitic acid (57.7%) and oleic acid (17.4%), while the leaves of the same cultivar recorded the highest amount of a-linolenic acid (52.5%). Finally, the highest content of linoleic acid was detected in the roots of cv. Pablo F1. Considering the classification of fatty acids, the leaves of beetroots and Swiss chard exhibited the highest levels of polyunsaturated fatty acids (PUFAs), ranging between 63.5 and 65.8%, mainly due to the contribution of C18:3n3 levels (47.8 to 52.5%) and C18:2n6c (12.61 to 17.6%), followed by saturated fatty acids (SFAs) (24.2 to 26.8%) and monounsaturated fatty acids (MUFAs) (9.4 to 9.9%). In this case, Swiss chard stood out among the rest in terms of its PUFA content, with high levels of oleic and α-linolenic acid (17.6 and 47.8%, respectively) (Figure 1). The classification of fatty acids in cv. Albina Vereduna roots showed the opposite trend, with SFAs being the main fatty acids (40.0 to 77.3%). In the case of cv. Burpee’s Golden roots, MUFAs were the second most abundant class of fatty acids (18.4%) followed by PUFAs (4.29%), while PUFAs recorded the highest amounts (42.8%) in cv. Pablo F1 roots, followed by SFAs (40.0%) and MUFAs (17.25%).

Stuiver et al. [46] analyzed the lipid composition of the roots and shoots of sugar beet and they also identified the prevalence of PUFAs in the leaves (65%) compared with the roots (57.9%). Similar results were found in the dehydrated beet leaves analyzed by Ebrahimi et al. [47], who reported the prevalence of linoleic, α-linolenic, palmitic, and oleic acids, as suggested in the present study. In the work of Mzoughi et al. [42], the fatty acid profile of chard was evaluated, determining the presence of 17 compounds, 16 of which are common to the variety studied in the present work. The levels of palmitic and linoleic acid were similar (19.6 and 17.6%, respectively), while the concentrations of α-linolenic acid (5.17%) and oleic acid (7.67%) were considerably lower than the results obtained.

#### 3.3.2. Tocopherols

**Figure 2:**
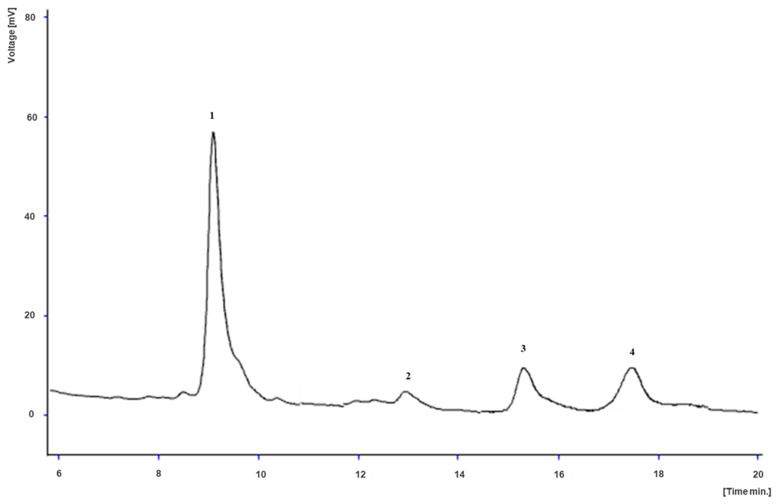
Tocopherol profile of *B. vulgaris* cv. Burpee’s Golden leaves, analyzed with HPLC-FL. 1: α-tocopherol; 2: *β*-tocopherol; 3: *γ*-tocopherol; 4: tocol, IS.

Table 2 also describes the results regarding tocopherols composition. The root samples presented only the α-tocopherol isoform (0.09 to 0.31 mg/100 g dw), while the α, *β* and *γ*-tocopherol isoforms were detected only in the leaf samples, except for the leaves of cv. Albina Vereduna, where *γ*-tocopherol was not detected. The content of total tocopherols in the leaves was significantly higher than that in the roots, due to the prevalence of the *α*-isoform (17.23 to 35.68 mg/100 g dw), whereas Swiss chard contained the lowest amounts of total tocopherols (18.05 mg/100 g dw). Moreover, the leaves of cv. Burpee’s Golden stood out for the highest content of *α*-tocopherol (35.68 mg/100 g dw), *γ*-tocopherol (3.10 mg/100 g dw) and consequently of total tocopherols (39.15 mg/100 g dw) (Figure 2). On the other hand, no significant differences were recorded between the tested leaf samples regarding *β*-tocopherol, except for the case of cv. Burpee’s Golden which recorded the lowest overall content (0.37 mg/100 g dw).

Xiao et al. [48], evaluated the composition and tocopherol content of different species of microgreens, including two varieties of *B. vulgaris* L. (e.g., “Bull’s Blood” and “Red”) and, similarly to the present study, identified *α*-tocopherol as the predominant isoform of vitamin E in both varieties (1.147 and 1.587 mg/100 g dw, respectively), as well as *γ*-tocopherol (“Bull’s Blood” with 0.31 mg/100 g dw and “Red” with 0.28 mg/100 g dw), whereas no *β*-tocopherol was identified. Samuolienė et al. [49] also evaluated the tocopherol content in the leaves of the “Bull’s Blood” variety and identified four isoforms (*α*-, *β*-, *γ*- and *δ*-) of tocopherols, with *β*- and *γ*-tocopherol being predominant, whereas *α*- and *δ*- isoforms were present at significantly lower levels. Santos et al. [50] determined the vitamin content in different species of leafy vegetables, where the presence of *α*-tocopherol was also reported in Swiss chard leaves.

### 3.4. Betalains

Betalains were detected only in leaf and root samples from cv. Pablo F1, roots from cv. Burpee’s Golden and Swiss chard leaves, with a total of 7 betacyanins and 1 betaxanthin being identified (Table 3). The compounds in peaks 1 and 2 (λmax = 534/532) presented the molecular ion [M+H]^+^ at *m*/*z* 551 and, in the MS^2^ spectrum, a characteristic fragment at *m*/*z* 389 [M+H–162]^+^, which was formed by the loss of glucose molecule resulting in the presence of the aglycones [betanidin+H]^+^ and [isobetanidine+H]^+^. Therefore, they were identified as betanin and isobetanin, respectively [51,52,53,54].

Furthermore, the compound detected in peak 5 (λmax = 534/532) was identified as neobetanin, due to its longer retention time in relation to betanin and isobetanin, and the presence of the molecular ion at *m*/*z* 549 (551–2 H), as well as by typical MS^2^ fragments generated at *m*/*z* of 387 (549–162) and 343 (387–44 (CO_2_)) [53,54].

Compounds in peaks 3b and 4 (λmax = 515 nm) were tentatively identified as decarboxy-betanin and decarboxy-isobetanin, respectively, due to the presence of molecular ions [M+H]^+^ at *m*/*z* 507 and an MS^2^ fragment at *m*/*z* 345 (507–162), these being the products of the decarboxylation of betanin and isobetanin (551–44). The compound in peak 6 corresponded to decarboxy-neobetanin, with [M+H]^+^ at *m*/*z* 505 and an MS^2^ fragment at *m*/*z* 343 (505–162) which resulted from the decarboxylation of neobetanin (549–44) [53,54].

The compound in peak 7 was tentatively identified as feruloyl-hexosyl-(iso)betanin, presenting the molecular ion [M+H]^+^ at *m*/*z* 889 and in the MS/MS spectrum, a fragment at *m*/*z* 389, characteristic of the presence of the aglycones [M+H–betanidine+H]^+^ or [isobetanidine+H]^+^; at *m*/*z* 713 [M+H–176]^+^, indicating the loss of the feruloyl group; and at *m*/*z* 727 [M+H–162]^+^, indicating the loss of a hexose [54].

The only betaxanthin identified was miraxanthin-V (peak 3a; λmax = 468; [M+H]^+^ *m*/*z* 347), which was recorded in small amounts in the root sample of cv. Burpee’s Golden and in Swiss chard leaves. The identification was based on the observation of a molecular ion at *m*/*z* 347 and its induced dissociation, which produced an informative spectrum of daughter ions dominated by the successive loss of carboxyl groups (base peak *m*/*z* 303 ([M+H−44 (CO_2_)]^+^ and a fragment at *m*/*z* 259 ([M+H–88 (2 × CO_2_)]^+^). It also showed fragmentations that corresponded to the loss of amino acids (*m*/*z* at 194 (347–153 (dopamine)), and a prominent ion at *m*/*z* 137, which represented deaminated dopamine (153-16 (NH_2_)) [52,55,56].

**Figure 3:**
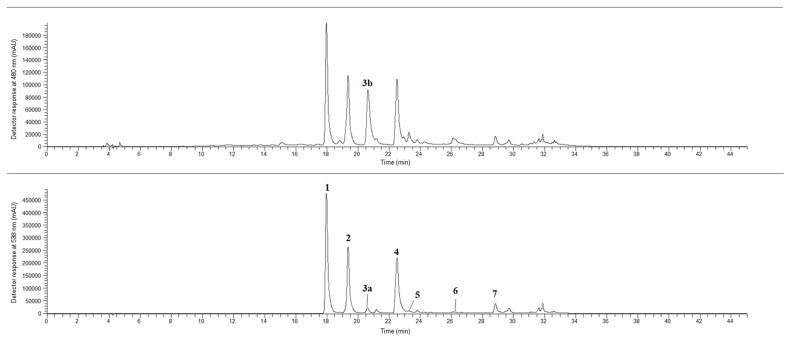
Betalain profile of Swiss chard leaves (*B. vulgaris* subsp. *cicla* var. *flavescens*) analyzed with LC-MS/MS. The peaks are identified in Table 3.

The most abundant compounds were decarboxy-isobetanin (Table 3, peak 4), especially in the roots of cv. Pablo F1 (18.18 mg/g dw), as well as betanin and isobetanin (Table 3; peaks 1 and 2, respectively) which were detected in the highest amounts in Swiss chard leaves (Figure 3) (3.86 and 2.35 mg/g dw for betanin and isobetanin, respectively). Sawicki et al. [57], analyzed the betalain profile of the roots of 13 beet varieties from Poland (NOE1, NOE2, NOE6, NOE8, 1A, 3A, 2B, 4B, SKR, SKB, Czarnota, Rywal and Crosby) and reported the same betacyanins identified in the present work. However, miraxanthin-V was not identified, while other betaxanthins, such as vulgaxanthin I, II, III, and miraxanthin II and III, were suggested in that study. Slatnar et al. [54] also evaluated the betalain content in different parts (peel, pulp and petioles) of two varieties of red beet (cv. Pablo F1 and cv. Taunus F1) and a variety of yellow beet (cv. Boldor). The authors suggest that, in the red varieties, the identified betacyanins were betanin, isobetanin, neobetanin, decarboxy-betanin and feruloylhexosyl-betanin, while betaxanthins included vulgaxanthin-I and indicaxanthin; in the yellow variety only the presence of betaxanthins was reported [54].

Kugler et al. [56] also suggested the presence of miraxanthin-V when they evaluated the betaxanthin profile in red (var. conditiva Alef.) and yellow (var. conditiva Alef. cv. Burpee’s Golden) beet varieties, and a yellow Swiss chard variety (*B. vulgaris* subsp. *cicla* cv. ’Bright Lights’). Other betaxanthins also identified included vulgaxanthin I, II, IIII and IV; miraxanthin II and III; and portucalaxanthin II and III, while a total of 25 compounds were identified in all of the samples.

### 3.5. Phenolic Compounds

The results regarding the identification and quantification of phenolic compounds detected in leaf and root samples are presented in Table 4 and Table 5, respectively. Nineteen compounds were identified in the leaves (Table 4), the majority of which include five compounds that were identified as *O*-glycosylated derivatives of vitexin (*O*,*C*-glycosylated flavones). These compounds, as well as vitexin itself (*C*-glycosylated derivative of the flavone apigenin), showed the presence of a characteristic fragment at *m*/*z* 293 (which represents the aglycone apigenin + 41 − 18 u), in addition to losses of 120 u, characteristic of the loss of the hexoside in *C*-glycosylated flavones. However, the identified compounds also presented abundant fragments related to the fragmentation of sugars plus 18 u (H_2_O), resulting in losses of 180 u (162 + 18) for hexosides, 150 u (132 + 18) for pentoxides, and 164 (146 + 18) for deoxyhexosides, generating abundant fragments at *m*/*z* 413 and *m*/*z* 455, respectively. According to Ferreres et al. [58] and Hegazi et al. [59], this additional loss of 18 u is characteristic of *O*-glycosylated flavones in position 2″ of the *C*-glycosylated sugar. Peak 9 (λmax = 336 nm; [M-H]^−^ *m*/*z* 593) showed a fragment at *m*/*z* 413, referring to the presence of a hexoside as sugar (loss of 180 u), while in peak 10a (λmax = 334 nm; [M-H]^−^ *m*/*z* 563) the loss of 150 u indicates the loss of a pentose, these being tentatively identified as vitexin-2″-*O*-hexoside and vitexin-2″-*O*-pentoside, respectively. The compound detected in peak 10b (λmax = 337 nm; [M-H]^−^ *m*/*z* 577) was identified as vitexin-2″-*O*-deoxyhexoside, due to the presence of a fragment at *m*/*z* 413 that indicated the loss of a deoxyhexoside (164 u). For peaks 12a (λmax = 347 nm; [M-H]^−^ *m*/*z* 679) and 13 (λmax 347 nm; [M-H]^−^ *m*/*z* 679), the presence of fragments at *m*/*z* 455 (attributed to losses of 180 u and 150 u) in addition to fragments at *m*/*z* 593 and 563 (loss of 86 u), which indicate the presence of the malonyl group in its structure, led to the identifications of these compounds as vitexin malonyl-2″-*O*-hexoside and vitexin malonyl-2″-*O*-pentoside, respectively [59,60,61,62,63,64].

Furthermore, three isorhamnetin derivatives were identified. The compound in peak 11 (λmax = 352 nm; [M-H]^−^ *m*/*z* 639) lost a fragment of 324 u, which indicates the loss of two hexoses (2 × 162 u), giving rise to the fragment at *m*/*z* 315 (characteristic of the isorhamnetin aglycone), being tentatively identified as isorhamnetin-*O*-dihexoside. Compound 12b (λmax = 347 nm; [M-H]¯ *m*/*z* 609) was classified as isorhamnetin-*O*-pentosyl-hexoside, whereby the [M-H]¯ ion at *m*/*z* 609 loses a fragment of 294 u, which refers to the loss of a hexose + pentose (162 + 132). Peak 14 (14; λmax = 367 nm; [M-H]¯ *m*/*z* 623) shows the loss of a fragment at 308 *m*/*z*, which is indicative of the presence of a rutinoside (deoxyhexoside-hexoside; 146 (deoxyhexoside) + 162 (hexose)), being tentatively identified as isorhamnetin-*O*-rutinoside [59,65,66].

In addition, two quercetin derivatives were identified. In the compound in peak 7 (λmax = 351 nm; [M-H]¯ *m*/*z* 625), the presence of characteristic fragment at *m*/*z* 301 indicates the loss of 324 u, corresponding to two hexoses (2 × 162 u), which was identified as quercetin-*O*-dihexoside, while peak 8 (λmax = 342 nm; [M-H]¯ *m*/*z* 595)was identified as quercetin-*O*-pentosyl-hexoside, as it demonstrated the loss of a 294 u fragment, which refers to hexose + pentose (162 + 132) [65,67].

The compound detected in peak 1 (λmax = 315 nm; [M-H]¯ *m*/*z* 355) was identified as *p*-coumaroyl-hexaric acid, being present in both root and leaf samples. The molecular ion loses *p*-coumaric acid [M-H-146]¯ during fragmentation (*p*-coumaric acid is also characterized by the presence of characteristic fragments at *m*/*z* 163 and *m*/*z* 119), leaving hexaric acid (*m*/*z* 209) and its dehydrated derivative (*m*/*z* 191) as the main fragments [65,67,68].

At peaks 3b and 5a, two isomers of the compound were tentatively identified as medioresinol (1 and 2, respectively; λmax = 230 nm; [M-H]¯ *m*/*z* 387). Medioresinol is a furofuran-type lignan that has antimicrobial and antiparasitic activity, already described as characteristic compounds in the species of *B. vulgaris* [69,70,71,72,73,74].

Derivatives of sinapic and ferulic acids were also identified, some of which are common among leaves and roots, such as sinapic acid-rhamnoside 1 and 2 (Table 4: peak 2/Table 5: peak 4 and 5; λmax = 313 nm; [M-H]¯ *m*/*z* 369), which exhibit a characteristic peak of sinapic acid at *m*/*z* 223, indicating the loss of a rhamnose (146 u); and ferulic acid-dihexoside (Table 4: peak 5b/Table 5: peak 6b; λmax = 326 nm; [M-H]¯ *m*/*z* 517), which presented a base peak characteristic of ferulic acid at *m*/*z* 193, indicating the loss of two hexoses (324 u), in addition to the *m*/*z* fragments 175 (193–18 (H_2_O)) and *m*/*z* 134 [66,75,76].

Some ferulic acid derivatives were found only in leaf samples, such as isomers 1 and 2 of ferulic acid-hexoside (Table 4: peaks 3a and 4; λmax = 330 nm; [M-H]¯ *m*/*z* 355), which demonstrated the characteristic peak of ferulic acid at *m*/*z* 193, indicating the loss of hexose (162 u), and fragments at *m*/*z* 175 and 134. Thus, the ferulic acid derivative present in peak 6a was also identified as butanetetraol-(feruloyl)-hexoside (paederol B; λmax = 319 nm; [M-H]^−^ = 459), due to the presence of the same characteristic fragments [59,77,78,79,80,81].

Another derivative of sinapic acid found only in leaves was identified as sinapic acid-hexoside, (Table 4: peak 6b; λmax = 319 nm; [M-H]^−^ *m*/*z* 385), which has a molecular ion at *m*/*z* 385 and the fragmentation of which gave rise to a peak at *m*/*z* 223, characterized by the loss of the hexoside residue (−162 u) [59,82,83].

The roots presented lower variability of phenolic compounds, with eight phenolic acids being identified in total, all of which were derived from ferulic, sinapic, *p*-coumaric and caffeic acids (Table 5). In addition to the compounds already described for leaves, two isomers of feruloyl-sinapic acid were also identified (peaks 6a and 7; λmax = 326 nm; [M-H]^−^ *m*/*z* 399), which demonstrated fragments that are characteristic of both sinapic acid at *m*/*z* 223, as well as ferulic acid at *m*/*z* 193 and 175 and which indicate the respective losses of each of these portions. In the roots, two isomers of a compound derived from caffeic acid were also detected (peaks 2 and 3; λmax = 300 nm; [M-H]^−^ *m*/*z* 341), characterized by the molecular ion at *m*/*z* 341 that gave rise to the base peak at *m*/*z* 179, corresponding to the loss of the hexoside ([M-H-162]^−^), as well as characteristic MS^2^ fragments at *m*/*z* 161 [M-H-hexose-H_2_O]^−^, *m*/*z* 135, *m*/*z* 153 [M-H-hexose-HC≡CH-H_2_O]^−^ and *m*/*z* 143 [M-H-hexose-2×H_2_O]^−^. The detected compounds were identified as caffeic acid-hexoside 1 and 2, respectively [84,85,86,87,88,89].

**Table 4:** Retention time (Rt), wavelengths of maximum absorption (λmax), mass spectral data, relative abundances of fragment ions, tentative identification and quantification of the phenolic compounds of the studied hydroethanolic extracts from *Beta vulgaris* leaves.

Leaves									
Peak n.°	Rt (min)	λmax (nm)	[M–H]^−^ (*m*/*z*)	MS^2^ Fragments (*m*/*z*)	Tentative Identification	Quantification (mg/g of Extract)			
						*Beta vulgaris* cv. Albina Vereduna	*Beta vulgaris* cv. Burpee’s Golden	*Beta vulgaris* cv. Pablo F1	*Beta vulgaris* subsp. cicla var. Flavescens
1	4.42	315	355	MS^2^ (355): 209 (100), 191 (90), 193 (87), 147 (56), 163 (29), 178 (25), 173 (10), 202 (10), 119 (8)	*p*-Coumaroyl hexaric acid [65,67,68]	tr	nd	nd	nd
2	5.69	-	369	MS^2^ (369): 129 (100), 223 (84), 205 (72), 163 (41), 119 (18), 202 (7)	Sinapic acid rhamnoside 1 [76]	0.050 ± 0.001 a	nd	nd	nd
3a *	6.18	-	355	MS^2^ (355): 193 (100), 178 (36), 134 (30), 149 (28), 194 (4), 175 (1)	Ferulic acid hexoside 1 [77,78,79,80]	tr	0.050 ± 0.002 a	0.030 ± 0.002 b	tr
3b *	6.18	-	387	MS^2^ (387): 163 (100), 387 (68), 119 (8), 388 (5), 164 (4), 207 (2), 369 (2)	Medioresinol 1 [69,70,72,73]	0.520 ± 0.002 c	0.540 ± 0.006 b	0.550 ± 0.001 a	0.490 ± 0.004 d
4	6.53	330	355	MS^2^ (355): 175 (100), 160 (21), 193 (9), 134 (6), 191 (3)	Ferulic acid hexoside 2 [77,78,79,80]	nd	nd	nd	0.39 ± 0.03 a
5a *	6.60	230	387	MS^2^ (387): 163 (100), 387 (68), 119 (8), 388 (5), 164 (4), 207 (2), 369 (2)	Medioresinol 2 [69,70,72,73]	0.530 ± 0.002 c	0.88 ± 0.02 a	0.74 ± 0.01 b	0.890 ± 0.004 a
5b *	6.60	326	517	MS2 (517): 175 (100), 193(98), 235 (32), 134 (26), 149 (9), 337 (8)	Ferulic acid dihexoside [66,75]	tr	0.46 ± 0.01 a	0.040 ± 0.002 c	0.100 ± 0.003 b
6a *	6.88	319	459	MS^2^ (459): 175 (100), 193 (46), 160 (12), 134 (10)	Ferulic acid derivative	0.14 ± 0.01 c	0.35 ± 0.01 a	0.25 ± 0.01 b	0.020 ± 0.001 d
6b *	6.88	319	385	MS^2^ (385): 223 (100), 208 (70), 164 (38), 179 (27), 149 (7), 205 (3), 193 (3)	Sinapic acid hexoside [82,83]	0.240 ± 0.008 a	nd	nd	0.110 ± 0.002 b
7	9.31	257/340/351	625	MS^2^ (625): 300 (100), 301 (61), 625 (31)	Quercetin dihexoside [67]	0.170 ± 0.003 c	0.170 ± 0.003 c	0.37 ± 0.01 a	0.250 ± 0.005 b
8	11.04	267/302/342	595	MS^2^ (595): 300 (100), 301 (54), 595 (34)	Quercetin pentosyl-hexoside [65]	0.28 ± 0.01 b	0.230 ± 0.002 c	0.38 ± 0.02 a	0.090 ± 0.003 d
9	11.27	214/269/336	593	MS^2^ (593): 293 (100), 413 (38), 311 (9), 294 (5), 414 (2), 473 (1)	Vitexin hexoside [66]	2.81 ± 0.02 b	0.55 ± 0.04 d	3.2 ± 0.1 a	0.89 ± 0.04 c
10a *	12.57	215/269/334	563	MS^2^ (563): 293 (100), 413 (36), 311 (11), 294 (6), 201 (4), 414 (3), 341 (1)	Vitexin pentoside [64,66]	2.87 ± 0.09 c	8.3 ± 0.4 a	8.5 ± 0.5 a	4.87 ± 0.03 b
10b *	12.57	214/269/337	577	MS2 (577): 293 (100), 413 (43), 311 (16), 294 (4), 457 (3), 341 (3)	Vitexin deoxyhexoside [73]	nd	nd	nd	5.0 ± 0.2a
11	13.9	204/256/352	639	MS^2^ (639): 315 (100), 314 (30), 300 (7), 639 (6), 316 (4), 299 (3), 357 (1)	Isorhamnetin dihexoside [66]	3.29 ± 0.05 c	3.5 ± 0.2 b	5.2 ± 0.3 a	1.8 ± 0.1 d
12a *	14.99	258/266/347	679	MS^2^ (679): 293 (100), 455 (44), 311 (11), 203 (6), 575 (6), 635 (1), 593 (1)	Vitexin malonyl-hexoside [66]	4.8 ± 0.2 a	2.32 ± 0.06 b	4.7 ± 0.2a	1.24 ± 0.02 c
12b *	14.99	258/266/347	609	MS^2^ (609): 315 (100), 314 (36), 609 (8), 300 (6)	Isorhamnetin hexosyl-pentoside [65]	4.84 ± 0.05 a	2.34 ± 0.07 b	4.9 ± 0.2 a	nd
13	15.99	216/269/334	649	MS2 (649): 293 (100), 455 (38), 311 (13), 545 (7), 294 (5), 341 (2), 605 (1), 563 (1)	Vitexin malonyl-pentoside [66]	2.37 ± 0.04 d	7.0± 0.3 a	5.9 ± 0.3 b	4.0 ± 0.3 c
14	16.64	267/338/367	623	MS^2^ (623): 315 (100), 314 (45), 623 (12), 300 (7)	Isorhamnetin rhamnoside-hexoside [65]	0.280 ± 0.003 b	nd	0.41 ± 0.02 a	nd
					Total phenolic acids	0.92 ± 0.01 a	0.86 ± 0.005 b	0.32 ± 0.01 d	0.62 ± 0.02c
					Total flavonoids	21.7 ± 0.4 c	25 ± 1 b	34 ± 2 a	18.2 ± 0.6 d
					Total phenolic compounds	23.2 ± 0.4 c	26.8 ± 0.9 b	35 ± 2 a	20.1 ± 0.7 d

*—Correspond to coeluted compounds in the peak of the same number; tr—traces; nd—not detected. Statistically significant differences (*p* < 0.05) between samples were assessed by a one-way ANOVA, using Tukey’s significant difference (HSD), and are indicated by different letters.

**Table 5:** Retention time (Rt), wavelengths of maximum absorption (λmax), mass spectral data, relative abundances of fragment ions, tentative identification and quantification of the phenolic compounds of the studied hydroethanolic extracts from *Beta vulgaris* roots.

Roots								
Peak n.°	Rt (min)	λmax (nm)	[M–H]^–^ (*m*/*z*)	MS^2^ Fragments (*m*/*z*)	Tentative Identification	Quantification (mg/g of Extract)		
						*Beta vulgaris* cv. Albina Vereduna	*Beta vulgaris* cv. Burpee’s Golden	*Beta vulgaris* cv. Pablo F1
1	4.42	315	355	MS^2^ (355): 209 (100), 191 (90), 193 (87), 147 (56), 163 (29), 178 (25), 173 (10), 119 (8)	*p*-Coumaroyl hexaric acid [65,67,68]	tr	tr	nd
2	4.60	296	341	MS^2^ (341): 119 (100), 179 (47), 143 (29), 161 (24), 341 (24), 149 (20), 135 (3)	Caffeic acid-hexoside 1 [85,87]	0.060 ± 0.002 b	0.060 ± 0.002 a	nd
3	4.95	300	341	MS2 (341): 153 (100), 119 (19), 179 (19), 143 (7), 161 (6), 341 (5), 149 (3), 135 (1)	Caffeic acid-hexoside 2 [85,87]	0.050 ± 0.001 b	0.060 ± 0.001 a	nd
4	5.68	313	369	MS2 (369): 129 (100), 223 (77), 205 (69), 163 (42), 119 (39), 161 (8)	Sinapic acid rhamnoside 1 [76]	0.020 ± 0.002 b	tr	0.040 ± 0.003 a
5	6.23	300	369	MS2 (369): 129 (100), 223 (80), 205 (70), 163 (47), 119 (19), 161 (10)	Sinapic acid rhaminoside 2 [76]	tr	tr	nd
6a *	6.60	326	399	MS2 (399): 129 (100), 205 (48), 223 (44), 193 (37), 134 (14), 161 (6), 175 (6)	Feruloyl sinapic acid 1 [86]	0.020 ± 0.001 c	0.030 ± 0.001 b	0.060 ± 0.002 a
6b *	6.60	326	517	MS2 (517): 175 (100), 193(83), 235 (27), 134 (21), 149 (9), 337 (8)	Ferulic acid dihexoside [66,75]	tr	tr	tr
7	7.34	323	399	MS2 (399): 129 (100), 205 (48), 193 (48), 223 (37), 134 (17), 161 (8), 175 (3)	Feruloyl sinapic acid 2 [86]	tr	tr	0.020 ± 0.002 a
					Total phenolic acids	0.150 ± 0.001 a	0.150 ± 0.002 a	0.130 ± 0.003 b
					Total phenolic compounds	0.150 ± 0.001 a	0.150 ± 0.002 a	0.130 ± 0.003 b

*—Correspond to coeluted compounds in the peak of the same number; tr—traces; nd—not detected. Statistically significant differences (*p* < 0.05) between samples were assessed by a one-way ANOVA, using Tukey’s significant difference (HSD), and are indicated by different letters.

**Figure 4:**
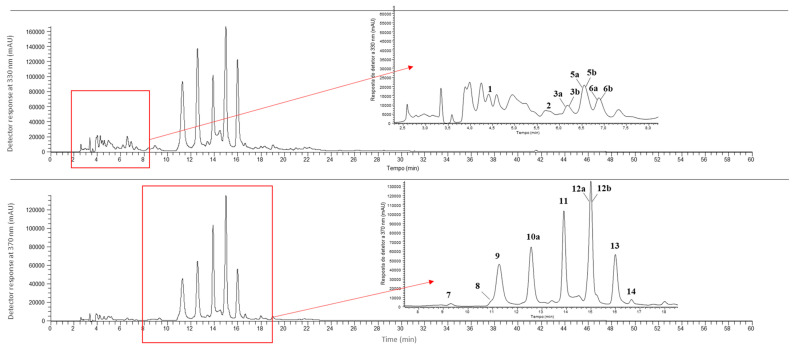
Phenolic profile of leaves from cv. Albina Vereduna analyzed with HPLC. The peaks are identified in Table 4.

**Figure 5:**
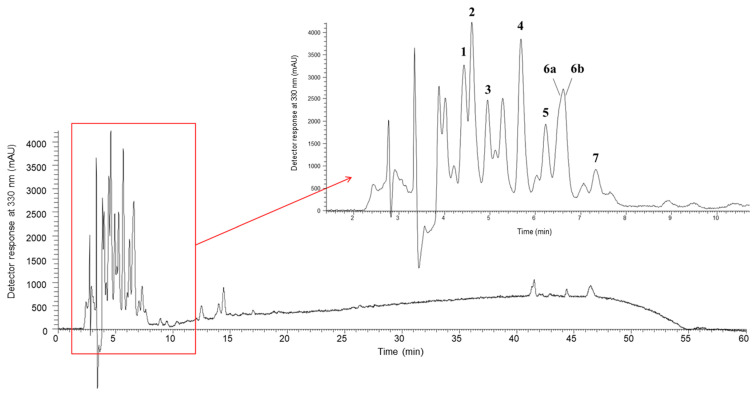
Phenolic profile of roots from cv. Albina Vereduna analyzed with HPLC. The peaks are identified in Table 5.

Regarding the quantities of the detected compounds, the content of total phenolic compounds in the leaves varied from 19.2 to 34.3 mg/g of extract, while the most abundant compounds were vitexin-2″-*O*-pentoside (peak 10a; 4.87 to 8.5 mg/g of extract), vitexin malonyl-2″-O-pentoside (peak 13; 2.37 to 7.0 mg/g of extract), isorhamnetin-*O*-dihexoside (peak 11; 3.29 to 5.2 mg/g of extract) and isorhamnetin-*O*-pentosyl-hexoside (peak 12b; 2.34 to 4.9 mg/g of extract). Moreover, flavonoids were the prevalent class of polyphenols in all of the studied leaf samples. The beetroot leaves demonstrated higher content of flavonoids, and consequently of polyphenols, than Swiss chard, with the leaves of cv. Albina Vereduna (Figure 4) having the highest content of phenolic compounds. The roots recorded lower levels of total phenolic compounds (0.130 to 0.150 g/100 g of extract), with a total of eight compounds identified, all of them identified as phenolic acids. The most abundant compounds were caffeic acid hexosides 1 and 2 (Table 5; peaks 2 and 3, respectively) which were detected only in the roots of cv. Albina Vereduna (Figure 5) (0.060 and 0.05 mg/g of extract, for caffeic acid hexosides 1 and 2, respectively) and cv. Burpee’s Golden (0.060 mg/g of extract for both compounds), while in the case of cv. Pablo F1 the most abundant compound was feruloyl-sinapic acid 1 (Table 5; peak 6a, 0.060 mg/g of extract). The presence of flavonoid glucosides derived from apigenin (vitexin), isorhamnetin and quercetin, as well as derivatives of phenolic acids such as *p*-coumaric, caffeic, ferulic and sinapic, together with derivatives of quercetin and isorhamnetin and flavones (e.g., vitexins) have already been reported in the roots and leaves of beets, as, for example, in the works of Vissers et al. [66] and Hegazi et al. [59], who evaluated the profile of phenolic compounds in the beet leaves of the Arrival and Rubra varieties, respectively.

Gawlik-Dziki et al. [20] analyzed the content of phenolic acids present in the leaves of two varieties of beetroot (red and white) and two varieties of Swiss chard (red (“rhubarb chard”) and white) from Poland, and reported the presence of *p*-coumaric, ferulic, and sinapic acids, in addition to other compounds, such as syringic, salicylic and caffeic acid, the latter being identified only in the roots of the Albina Vereduna and Burpee’s Golden varieties. The results obtained from the referred study also demonstrate that the red variety of Swiss chard has the highest content of phenolic acids. In contrast to the results obtained in the present work, Płatosz et al. [90] analyzed the extracts from fermented beet root juices, and, apart from caffeic, ferulic, sinapic and *p*-coumaric acids, they also detected other acids, such as protocatechuic, syringic, chlorogenic, and flavonoids, such as apigenin, vitexin, quercetin, kaempferol, rutin and luteolin, which were not detected in our study.

### 3.6. Bioactive Properties

#### 3.6.1. Antioxidant Activity

The results related to the evaluation of antioxidant activity are presented in Table 6. It was possible to identify significant differences between the samples, with better antioxidant activity being recorded in the leaves than in the roots, especially the leaves of cv. Pablo F1, where the highest overall antioxidant activity was recorded (EC_50_ of 0.35 mg/mL). In addition, the roots of cv. Pablo F1 recorded the highest antioxidant activity among the tested root samples (EC_50_ of 1.13 mg/mL), indicating the high potential of this particular genotype. Finally, all of the leaf samples recorded higher antioxidant activity than the positive control used (Trolox; EC_50_ of 0.88 mg/mL).

Rey et al. [91] evaluated the antioxidant activity of red beet methanolic extracts using the TBARS methodology, revealing a high antioxidant action (at a concentration of 1.35 mg of MDA/g of meat). There are other methods widely used to evaluate the antioxidant potential of extracts. However, although they are effective in evaluating this property, the vast majority are chemical tests based on the elimination of free radicals, meaning they do not allow an effective comparison with the methodology applied in the present study. For example, Gawlik-Dziki et al. [20] analyzed the antioxidant activity of methanolic extracts from leaves of two cultivars of beetroot (white and red) and Swiss chard (rhubarb and white chard) from Poland using the ABTS test and observed that rhubarb chard and leaves of red beetroot showed high antioxidant capacity (EC_50_ = 9.91 and 11.9 g mg/mL, respectively). The same authors tested two more assays (e.g., hydroxyl radical scavenging ability and chelating power) and suggested that beetroot leaf extracts showed the highest antioxidant power.

Sawicki et al. [57], who used four different assays (e.g., photochemiluminescence (PCL) method under lipophilic (ACL) and hydrophilic conditions (ACW), ABTS and DPPH) for the determination of the antioxidant activity, reported a wide range of values, while suggesting that the extracts of the thirteen red beetroot varieties tested showed the highest efficiency for the ABTS assay. In addition, the same authors suggested that the peels of the roots had the highest antioxidant capacity compared with the inner parts (rings) of the root, being positively correlated with the betalain content of each individual root part. Similar results have been reported by Ninfali and Angelino [5] who recorded a high variation in the antioxidant activity of the leaves, roots and seeds of Swiss chard and beetroot, with leaves showing the highest ORAC values, while similar results are reported by Wruss et al. [40], who tested seven different red beetroot varieties. In contrast, Salamatullah et al. [92] did not observe significant differences in the reducing power of peels and pulp of beet roots, whereas they suggested a significant impact of the extraction solvent (methanol, 50% of methanol and water) on the reducing power of the tested root parts. In the study of Mzoughi et el. [42], the evaluation of the antioxidant activity of leaves of wild Swiss chard showed significant activity for all of the implemented assays (DPPH, ABTS, reducing power, β-carotene bleaching and TBARS) and the authors attributed these activities to the high content of polyphenols.

Moreover, Asadi and Khan [37] tested the antioxidant activity of dried beetroot leaf powder using the DPPH method, aiming to incorporate them in cookies at different amounts and reported values of 43.84%. Gennari et al. [60] also evaluated the antioxidant potential using the oxygen radical absorbance capacity (ORAC) assay of Swiss chard seeds and suggest an antioxidant activity of 4269.76 mMol TE/g for the dry extract. Bavec et al. [43] used the DPPH assay to evaluate the antioxidant activity of the roots of red beetroot (cv. Rote Kugel) cultivated under four different cultivation systems and report values between 0.823 μMTE/g FW to 1.270 μMTE/g FW of Trolox equivalents.

#### 3.6.2. Antimicrobial Activity

Table 7 presents the results regarding the antimicrobial activity of the hydroethanolic extracts of the tested root and leaf samples against three gram-positive and five gram-negative pathogenic bacteria and two fungi. All samples exhibited antibacterial capacity against the strains tested, except for the bacteria *Enterobacter cloacae* and *Pseudomonas aeruginosa*, where no activity was demonstrated. The leaf samples of cv. Albina Vereduna were the only ones to show activity against the analyzed fungi, standing out for their overall antimicrobial capacity. Swiss chard has also exhibited significant antibacterial activity, especially against the gram-negative bacteria *Staphylococcus aureus*. In contrast, the roots of cv. Burpee’s Golden showed low antimicrobial capacity.

In the study carried out by Salamatullah et al. [92], the antimicrobial activity of extracts (methanolic and aqueous) of the pulp and peel of red beetroot (var. rubra) were evaluated against the strains *Staphylococcus aureus*, *Listeria monocytogenes*, *Pseudomonas aeruginosa*, *Escherichia coli* and *Candida albicans* and the MICs of the extracts were determined using the microdilution method. The results showed that all of the extracts showed low to moderate antimicrobial activity against the tested pathogens. Vulić et al. [93] also evaluated the antimicrobial properties of the extracts of beet residues obtained after juice preparation, by using microorganism cultures from food and water, including six gram-negative bacteria, eight gram-positive bacteria, two yeasts and two molds. The obtained results showed that all of the gram-negative bacteria and some gram-negative bacteria were inhibited by the tested extract, whereas no effects against the tested fungi were demonstrated.

Chaari et al. [94] analyzed the antibacterial potential of different extracts (acetonitrile, ethanol, and water in different proportions) of beet leaves against a gram-positive bacteria (*S. aureus*) and two gram-negative bacteria (*Salmonella enterocolitica* and *E. coli*) and the optimized leaf extract was able not only to inhibit, but also to present bactericidal activity against all pathogens studied. The study of Ahmadi et al. [95] evaluated the antimicrobial activity of methanolic aqueous extracts and essential oils from red beet leaves, where it was found that the methanolic extract and the essential oils had the greatest capacity to inhibit the growth of *E. coli*, *B. cereus*, *S. aureus*, *S. enteritidis*, while they recorded antifungal activity against *Byssochlamys fulva*, *Aspergillus niger*, *Geotrichum* spp., *Alternaria* spp. and *Fusarium* spp. Yerubay et al. [96] performed the extraction of *B. vulgaris* seeds by using the supercritical CO_2_ method and suggested moderate antimicrobial efficacy against the strains of *S. aureus*, *Bacillus subtilis*, *E. coli*, and *Proteus vulgaris* and the yeast of *C. albicans.*

## 4. Conclusions

The growing awareness of the beneficial effects of a healthier diet and lifestyle has increased the interest in new/alternative dietary sources of functional compounds. Given this, in the present study we assessed the chemical composition and biological properties of the roots and leaves of different varieties of beetroot (*B. vulgaris* subsp. *vulgaris*) with different root colors, as well as a variety of Swiss chard (*B. vulgaris* subsp. *cicla*). Our results highlight the high nutritional value of beetroot leaves compared not only with the respective roots but also to Swiss chard leaves, especially regarding their phenolic compound profile and the antioxidant activity. Although the consumption and properties of beetroot roots are highly appreciated, beetroot leaves can also be considered a valuable dietary source of macronutrients and functional ingredients. Therefore, they could be further valorized and introduced into diets, as they can contribute to food diversification, ensuring the intake of a wide range of essential nutrients and bioactive compounds that are associated with significant health benefits.

## Figures and Tables

**Table 1:** Proximal composition, energetic value and hydrophilic compounds of *Beta vulgaris* samples (mean ± SD, *n = 3*).

	*Beta vulgaris* cv. Albina Vereduna		*Beta vulgaris* cv. Burpee’s Golden		*Beta vulgaris* cv. Pablo F1		*Beta vulgaris* subsp. cicla var. Flavescens
	Leaves	Roots	Leaves	Roots	Leaves	Roots	Leaves
Proximal composition	(g/100 g dw)						
Fat	3.30 ± 0.06 b	1.12 ± 0.03 d	3.28 ± 0.02 b	0.48 ± 0.06 f	4.01 ± 0.04 a	0.55 ± 0.05 e	2.18 ± 0.10 c
Proteins	11.84 ± 0.06 c	5.46 ± 0.04 g	12.23 ± 0.06 a	6.22 ± 0.07 f	12.18 ± 0.04 b	7.32 ± 0.06 e	9.94 ± 0.01 d
Ash	13.46 ± 0.01 c	2.35 ± 0.05 g	17.77 ± 0.07 b	3.21 ± 0.03 f	20.99 ± 0.04 a	4.0 ± 0.1 e	11.91 ± 0.09 d
Dietary fibers (%)	34.2 ± 0.2 b	20.9 ± 0.2 d	33.9 ± 0.4 b	19.5 ± 0.6 e	37.3 ± 0.7 a	19.9 ± 0.1 e	33.1 ± 0.9 c
Carbohydrates	37.18 ± 0.05 d	70.2 ± 0.2 a	32.8 ± 0.3 e	70.6 ± 0.4 a	25.5 ± 0.5 f	68.3 ± 0.1 b	42.9 ± 0.6 c
Energy (kcal/100 g dw)	294.24 ± 0.5 e	354.4 ± 0.5 a	277.5 ± 0.7 f	350.5 ± 0.7 b	261.5 ± 0.8 g	347.0 ± 0.3 c	297.1 ± 0.8 d
Free sugars	(g/100 g dw)						
Frutose	2.8 ± 0.02 a	nd	0.97 ± 0.07 c	nd	0.45 ± 0.04 d	nd	1.57 ± 0.02 b
Glucose	5.65 ± 0.08 a	nd	3.84 ± 0.06 b	nd	2.24 ± 0.04 c	nd	3.5 ± 0.06 d
Sucrose	5.08 ± 0.04 e	35.93 ± 0.03 b	6.21 ± 0.04 d	33.94 ± 0.03 c	3.24 ± 0.04 g	40.79 ± 0.67 a	4.04 ± 0.08 f
Trehalose	0.69 ± 0.03 b	0.38 ± 0.01 d	0.82 ± 0.01 a	0.46 ± 0.03 c	0.7 ± 0.05 b	0.7 ± 0.03 b	0.41 ± 0.06 d
Total	11.4 ± 0.2 d	36.31 ± 0.03 b	10.87 ± 0.03 e	34.4 ± 0.06 c	6.2 ± 0.1 g	41.5 ± 0.6 a	7.96 ± 0.2 f
Organic acids	(g/100 g dw)						
Oxalic acid	2.13 ± 0.02 d	0.89 ± 0.02 f	2.89 ± 0.03 b	0.85 ± 0.03 f	3.78 ± 0.21 a	1.33 ± 0.01 e	2.37 ± 0.03 c
Quinic acid	1.61 ± 0.03 d	0.49 ± 0.04 g	3.15 ± 0.07 b	0.64 ± 0.04 f	3.97 ± 0.09 a	1.36 ± 0.01 e	2.54 ± 0.01 c
Malic acid	1.44 ± 0.01 c	0.34 ± 0.01 g	1.86 ± 0.04 b	0.76 ± 0.02 f	2.8 ± 0.04 a	0.95 ± 0.01 e	1.40 ± 0.01 d
Citric acid	1.42 ± 0.02 d	0.35 ± 0.02 g	2.02 ± 0.03 c	0.81 ± 0.03 f	2.67 ± 0.05 a	1.11 ± 0.02 e	2.2 ± 0.2 b
Succinic acid	5.2 ± 0.3 f	6.0 ± 0.2 e	6.7 ± 0.1 d	4.29 ± 0.01 g	9.7 ± 0.2 c	15 ± 0.3 a	12.4 ± 0.07 b
Fumaric acid	tr	tr	tr	tr	tr	tr	tr
Total	11.8 ± 0.4 e	8.1 ± 0.3 f	16.6 ± 0.3 d	7.4 ± 0.1 g	22.9 ± 0.2 a	19.7 ± 0.3 c	20.9 ± 0.3 b

Means in the same line followed by different Latin letters are significantly different according to Tukey’s honestly significant difference (HSD) test at *p* = 0.05; tr—traces; nd—not detected.

**Table 2:** Chemical composition with regard to the lipophilic compounds of *Beta vulgaris* samples (mean ± SD, *n* = 3).

	*Beta vulgaris* cv. Albina Vereduna		*Beta vulgaris* cv. Burpee’s Golden		*Beta vulgaris* cv. Pablo F1		*Beta vulgaris* subsp. *cicla* var. Flavescens
	Leaves	Roots	Leaves	Roots	Leaves	Roots	Leaves
Fatty Acids	Relative Percentage (%)						
C11:0	nd	3.57 ± 0.02	nd	0.55 ± 0.01	0.11 ± 0.01	0.47 ± 0.01	nd
C12:0	0.61 ± 0.01	0.49 ± 0.01	0.21 ± 0.01	0.75 ± 0.01	0.21 ± 0.01	0.41 ± 0.01	0.10 ± 0.01
C13:0	0.60 ± 0.01	nd	0.61 ± 0.04	nd	0.64 ± 0.04	0.054 ± 0.002	0.56 ± 0.02
C14:0	0.91 ± 0.02	0.83 ± 0.01	0.64 ± 0.01	0.90 ± 0.01	0.75 ± 0.02	0.69 ± 0.01	0.52 ± 0.01
C14:1	nd	nd	0.26 ± 0.01	nd	nd	nd	nd
C15:0	0.22 ± 0.01	1.05 ± 0.01	0.19 ± 0.01	1.4 ± 0.04	0.21 ± 0.01	0.76 ± 0.03	0.19 ± 0.01
C15:1	0.17 ± 0.02	nd	0.19 ± 0.01	nd	nd	nd	0.19 ± 0.01
C16:0	19.17 ± 0.05	42.6 ± 0.1	19.6 ± 0.3	57.7 ± 0.1	20 ± 0.2	29.3 ± 0.4	19.6 ± 0.5
C16:1	1.59 ± 0.02	nd	1.64 ± 0.08	nd	1.78 ± 0.04	0.61 ± 0.02	1.43 ± 0.03
C17:0	0.55 ± 0.01	1.99 ± 0.03	0.48 ± 0.02	7.14 ± 0.09	0.48 ± 0.03	1.87 ± 0.03	0.36 ± 0.03
C18:0	2.07 ± 0.02	4.9 ± 0.01	1.4 ± 0.08	2.74 ± 0.03	1.66 ± 0.01	2.55 ± 0.1	1.36 ± 0.03
C18:1n9c	7.5 ± 0.01	9.53 ± 0.03	7 ± 0.2	17.4 ± 0.1	7.5 ± 0.2	16.03 ± 0.04	7.67 ± 0.09
C18:2n6c	13.82 ± 0.01	21.37 ± 0.06	12.61 ± 0.07	2.95 ± 0.02	13.1 ± 0.1	37.2 ± 0.4	17.6 ± 0.1
C18:3n6	nd	1.99 ± 0.01	0.34 ± 0.01	1.03 ± 0.01	nd	nd	0.37 ± 0.02
C18:3n3	49.85 ± 0.03	2.7 ± 0.01	52.5 ± 0.5	0.31 ± 0.01	50.3 ± 0.7	5.53 ± 0.03	47.8 ± 0.1
C20:0	nd	nd	nd	0.82 ± 0.02	nd	nd	nd
C20:1	0.45 ± 0.01	nd	0.31 ± 0.01	0.78 ± 0.01	0.41 ± 0.01	0.61 ± 0.02	0.45 ± 0.02
C22:0	0.85 ± 0.02	2.39 ± 0.07	0.67 ± 0.05	1.15 ± 0.01	0.96 ± 0.02	1.08 ± 0.02	0.51 ± 0.01
C22:1	nd	nd	nd	0.32 ± 0.01	nd	nd	nd
C23:0	0.53 ± 0.01	1.71 ± 0.05	0.36 ± 0.01	1.23 ± 0.01	0.45 ± 0.01	1.00 ± 0.01	0.25 ± 0.01
C24:0	1.11 ± 0.01	4.89 ± 0.01	0.97 ± 0.06	2.92 ± 0.02	1.31 ± 0.01	1.8 ± 0.03	0.74 ± 0.03
SFA	26.62 ± 0.01 d	64.4 ± 0.1 b	25.1 ± 0.6 e	77.3 ± 0.2 a	26.8 ± 0.3 d	40 ± 0.5 c	24.2 ± 0.4 f
MUFA	9.71 ± 0.04 d	9.53 ± 0.03 e	9.4 ± 0.08 f	18.4 ± 0.2 a	9.7 ± 0.2 d	17.25 ± 0.01 b	9.9 ± 0.1 c
PUFA	63.67 ± 0.04 c	26.06 ± 0.08 e	65.5 ± 0.5 b	4.29 ± 0.03 f	63.5 ± 0.5 c	42.8 ± 0.5 d	65.8 ± 0.2 a
Tocopherols	(mg/100 g dw)						
*α*-Tocopherol	26.02 ± 0.05 c	0.22 ± 0.01 f	35.68 ± 0.03 a	0.09 ± 0.01 g	26.06 ± 0.01 b	0.31 ± 0.01 e	17.23 ± 0.09 d
*β*-Tocopherol	0.393 ± 0.003 a	nd	0.37 ± 0.02 b	nd	0.40 ± 0.01 a	nd	0.40 ± 0.01 a
*γ*-Tocopherol	nd	nd	3.10 ± 0.05 a	nd	0.42 ± 0.03 b	nd	0.42 ± 0.01 b
Total	26.41 ± 0.04 c	0.22 ± 0.01 f	39.15 ± 0.06 a	0.09 ± 0.01 g	26.88 ± 0.01 b	0.31 ± 0.01 e	18.05 ± 0.08 d

nd—not detected; SFA: saturated fatty acid; MUFA: monounsaturated fatty acid; PUFA: polyunsaturated fatty acid. Statistically significant differences (*p* < 0.05) between samples were assessed by a one-way ANOVA, using Tukey’s significant difference (HSD), and are indicated by different letters.

**Table 3:** Retention time (Rt), wavelengths of maximum absorption (λmax), mass spectral data, relative abundances of fragment ions, tentative identification and quantification of the betalains found in leaves and roots from the *Beta vulgaris* varieties studied.

Leaves									
						Quantification (mg/g of dw)			
Peak n.°	Rt (min)	λmax (nm)	[M-H]^+^ (*m*/*z*)	MS^2^ Fragments (*m*/*z*)	Tentative Identification	Leaves		Roots	
						*Beta vulgaris* cv. Pablo F1	*Beta vulgaris* subsp. cicla var. Flavescens	*Beta vulgaris* cv. Pablo F1	*Beta vulgaris* cv. Burpee’s Golden
1	18.00	534	551	MS^2^ (551): 389 (100), 390 (11), 302 (2)	Betanin [51,52,53,54]	0.820 ± 0.001 c	3.86 ± 0.02 a	0.90 ± 0.02 b	nd
2	19.40	532	551	MS^2^ (551): 389 (100), 390 (19)	Isobetanin [51,52,53]	0.800 ± 0.002 c	2.35 ± 0.04 a	1.48 ± 0.07 b	nd
3a *	20.65	468	347	MS^2^ (347): 303 (100), 137 (93), 259 (57), 106 (44), 194 (14), 164 (12)	Miraxanthin-V [51,55]	nd	0.350 ± 0.007 a	nd	0.14 ± 0.002 b
3b *	20.65	501	507	MS^2^ (507): 345 (100), 346 (20), 257 (3), 417 (3), 357 (2)	Decarboxy-betanin [54]	0.210 ± 0.001 c	0.350 ± 0.007 b	0.69 ± 0.05 a	nd
4	22.50	533	507	MS^2^ (507): 345 (100), 346 (12), 479 (1)	Decarboxy-isobetanin [54]	1.29 ± 0.02 c	2.54 ± 0.02 b	18.18 ± 0.03 a	nd
5	23.30	535	549	MS^2^ (549): 387 (100), 388 (20), 343 (1)	Neobetanin [53,54]	nd	0.300 ± 0.008 b	0.63 ± 0.06 a	nd
6	26.10	481	505	MS^2^ (505): 343 (100), 344 (11), 296 (3)	Decarboxy-neobetanin [53,54]	nd	0.170 ± 0.001 b	1.64 ± 0.11 a	nd
7	28.80	535	889	MS^2^ (889): 389 (100), 390 (14), 713 (3), 727 (3)	Feruloyl-hexosyl betanin [53]	nd	0.47 ± 0.04 a	nd	nd
					Total betacyanins	3.12 ± 0.02 c	10.0 ± 0.1 b	23 ± 1 a	-
					Total betaxanthins	-	0.350 ± 0.007 a	-	0.140 ± 0.002 b
					Total betalains	3.12 ± 0.02 c	10.4 ± 0.1 b	23 ± 1 a	0.140 ± 0.002 d

*—Correspond to coeluted compounds in the peak of the same number; nd—not detected. Statistically significant differences (*p* < 0.05) between samples were assessed by a one-way ANOVA, using Tukey’s significant difference (HSD), and are indicated by different letters.

**Table 6:** Antioxidant activity of the hydroethanolic extract from *Beta vulgaris* samples (mean ± SD, *n* = 3).

	*Beta vulgaris* cv. Albina Vereduna		*Beta vulgaris* cv. Burpee’s Golden		*Beta vulgaris* cv. Pablo F1		*Beta vulgaris* subsp. cicla var. Flavescens	
	Leaves	Roots	Leaves	Roots	Leaves	Roots	Leaves	Trolox
TBARS (EC_50_, mg/mL)	0.39 ± 0.06 f	3.06 ± 0.05 b	0.65 ± 0.02 e	12.3 ± 0.6 a	0.35 ± 0.02 g	1.13 ± 0.04 c	0.88 ± 0.02 d	0.054 ± 0.003

Statistically significant differences (*p* < 0.05) between samples were assessed by a one-way ANOVA, using Tukey’s significant difference (HSD), and are indicated by different letters.

**Table 7:** Antimicrobial activity (minimal inhibition concentration (MIC), minimal bactericidal concentration (MBC) and minimal fungicidal concentration (MFC), mg/mL) of the studied hydroethanolic extract from *Beta vulgaris* samples.

	*Beta vulgaris* cv. Albina Vereduna				*Beta vulgaris* cv. Burpee’s Golden				*Beta vulgaris* cv. Pablo F1				*Beta vulgaris* subsp. cicla var. Flavescens							
															Positive Controls					
Antibacterial Activity	Leaves		Roots		Leaves		Roots		Leaves		Roots		Leaves		Streptomicin 1 mg/mL		Methicilin 1 mg/mL		Ampicillin 10 mg/mL	
	MIC	MBC	MIC	MBC	MIC	MBC	MIC	MBC	MIC	MBC	MIC	MBC	MIC	MBC	MIC	MBC	MIC	MBC	MIC	MBC
Gram-negative bacteria																				
*Enterobacter cloacae*	10	>10	10	>10	10	>10	10	>10	10	>10	10	>10	10	>10	0.007	0.007	nt *	nt	0.15	0.15
*Escherichia coli*	5	>10	10	>10	10	>10	10	>10	5	>10	10	>10	5	>10	0.01	0.01	nt	nt	0.15	0.15
*Pseudomonas aeruginosa*	>10	>10	>10	>10	>10	>10	>10	>10	>10	>10	>10	>10	>10	>10	0.06	0.06	nt	nt	0.63	0.63
*Salmonella enterocolitica*	5	>10	5	>10	5	>10	>10	>10	10	>10	10	>10	5	>10	0.007	0.007	nt	nt	0.15	0.15
*Yersinia enterocolitica*	1.25	>10	1.25	>10	2.5	>10	>10	>10	5	>10	5	>10	2.5	>10	0.007	0.007	nt	nt	0.15	0.15
Gram-positive bacteria																				
*Bacillus cereus*	10	>10	>10	>10	5	>10	>10	>10	10	>10	2.5	>10	>10	>10	0.007	0.007	nt	nt	nt	nt
*Listeria monocytogenes*	10	>10	>10	>10	2.5	>10	5	>10	2.5	>10	5	>10	5	>10	0.007	0.007	nt	nt	0.15	0.15
*Staphylococcus aureus*	0.6	>10	1.25	>10	2.5	>10	5	>10	5	>10	1.25	>10	0.6	>10	0.007	0.007	0.007	0.007	0.15	0.15
Antifungal activity															Ketoconazole					
	MIC	MFC	MIC	MFC	MIC	MFC	MIC	MFC	MIC	MFC	MIC	MFC	MIC	MFC	MIC	MFC	-	-	-	-
*A. fumigatus*	>10	>10	5	>10	>10	>10	10	10	>10	10	>10	10	10	10	0.5	0.06	-	-	-	-
*A. brasiliensis*	10	>10	5	>10	10	>10	>10	>10	>10	>10	>10	>10	>10	>10	1	0.125	-	-	-	-

* nt: not tested.

## Data Availability

Data is contained within the article.
